# Supplementary material for: Harnessing flavonoids to control probiotic function: in situ application of a naringenin-responsive genetic circuit
Source: Microbiol Spectr. 2025 Apr 25;13(6):e02890-24. doi: 10.1128/spectrum.02890-24 (PMC12131760; doi:10.1128/spectrum.02890-24)
Supplement: Supplemental material — Detailed methodologies, additional figures, and supporting data for the characterization of the naringenin-responsive genetic circuit in probiotic E. coli Nissle 1917, both in vitro and in vivo. [file spectrum.02890-24-s0001.docx]

**Supplemental Material**

***Harnessing Flavonoids to Control Probiotic Function: In Vivo Application of a Naringenin-Responsive Genetic Circuit***

Brenno Wendler Miranda^1^, Lucas Henrique Junges^1^, Emanuel Maltempi de Souza^1^, Paula Santana Lunardi^2^, Marcelo Müller-Santos^1^

^1^ Postgraduate Program in Science (Biochemistry), Department of Biochemistry and Molecular Biology, Nitrogen Fixation Laboratory, Federal University of Paraná (UFPR), Curitiba, Brazil.

^2^ Postgraduate Program in Science (Biochemistry), Department of Biochemistry and Molecular Biology, Biological Oxidations Laboratory, Federal University of Paraná (UFPR), Curitiba, Brazil.

**Supplemental methodology**

**Bacterial strains and growth conditions**

*E. coli* Top10 (Invitrogen, USA) was used for plasmid construction and maintenance. For circuit characterization, *E. coli* Nissle 1917 (EcN) was utilized. The strain EcN *attλ::sfgfp* used in mice experiments was obtained as previously described (1). To generate EcN *attλ::sfgfp*, a *gfp* cassette carried by the pAH63 vector (Addgene #66073) was electroporated into EcN, along with the pINT-ts helper plasmid (Addgene #66076) to facilitate the integration of the cassette into the *attλ* site via site-specific recombination. Transformants carrying the integrated *gfp* cassette were selected on LB agar containing kanamycin (50 µg/mL). The pINT-ts helper plasmid was cured by growing the transformants at 42°C, a non-permissive temperature for pINT-ts replication, and selecting colonies on LB agar without antibiotics. All cultures were grown in LB medium (10 g/L tryptone, 5 g/L yeast extract, 10 g/L NaCl) or LB agar (LB medium with 15 g/L bacteriological agar) at 37°C and 180 rpm.

***In vitro* characterization of naringenin-responsive genetic circuits**

*E. coli* Nissle 1917 (EcN) strains carrying 114-*fdeR*-*sfgfp* or 114-*fdeR*-*nanoluc* plasmid were cultured in LB broth for 18 hours at 37°C with shaking at 180 rpm. Following the initial growth phase, cultures were diluted 1% (v/v) into 200 µL of fresh LB medium in a 96-well flat-bottom black polystyrene plate (Greiner) and incubated for three more hours. After incubation, flavonoids dissolved in DMSO were added to the wells at the desired concentrations, and the fluorescence was monitored over 15 h using a Tecan Infinite 200 plate reader (Tecan Life Sciences, Switzerland) at 37°C with 5 mm orbital shaking. Fluorescence readings were recorded every 10 minutes with excitation at 485 nm and emission at 535 nm, using a gain of 88. Optical density at 600 nm was simultaneously measured to track bacterial growth. For the 114-*fdeR*-*nanoluc* circuit, after 4 h of culture, 10 µL of the culture was sampled to measure the luminescence generated with the NanoGlo Luciferase Assay System™ (Promega, USA). Luminescence was measured using a Synergy LX microplate reader (BioTek) with a gain setting of 100.

For the data generated from the microplate readers, fluorescence values were normalized by optical density (OD_600_) using the following equation:

$$\left( \frac{\text{Flu}}{\text{OD}_{\text{600}}} \right)\text{=}\left( \frac{\text{Flu}_{\text{c}}\text{ }\text{- }\text{Flu}_{\text{m}}}{\text{OD}_{\text{c}}\text{ }\text{- }\text{OD}_{\text{m}}} \right)\text{-}\left( \frac{\text{Flu}_{\text{b}}\text{ }\text{-}\text{ Flu}_{\text{m}}}{\text{OD}_{\text{b}}\text{ }\text{- }\text{OD}_{\text{m}}} \right)\text{ eq. (1)}$$

where Flu_c_ is the fluorescence of the culture, OD_c_ is the OD_600_ of the culture, Flu_m_ is the fluorescence of the LB medium, OD_m_ is the OD_600_ of the LB medium, and Flu_b_ and OD_b_ represent the fluorescence and OD_600_ of a bacterial culture without the genetic circuits. The normalized fluorescence values were plotted against the input concentration to generate dose-response curves. A nonlinear regression of the data was performed using the SciPy package for Python (2) based on the Hill equation:

$$\frac{\text{Flu}}{\text{OD}_{600}}\text{ = }\text{k}^{\text{'}}\text{+ }\text{k}\left( \frac{\text{x}^{\text{n}}}{\text{x}^{\text{n}}\text{ +}{\text{ }\text{K}}^{\text{n}}} \right)\text{eq. }\text{(2)}$$

where *k*′ is the basal expression level, *k* is the maximum expression level, *x* is the input concentration, *K* is the Hill constant, representing the input concentration needed to reach half of the maximum output, and *n* is the Hill coefficient, indicating the cooperativity of the system.

***In situ* characterization of naringenin-responsive genetic circuits**

C57BL/6 mice were used for animal experiments, and all animals used in this study were bred and housed at the Animal Facility Complex of the Federal University of Paraná (UFPR), where maintenance and experimentation were conducted. The animals were housed in appropriately sized cages, with no more than five animals per cage at any time and no mixing of animals of different sexes. Water and food were provided *ad libitum* throughout the study. All procedures were approved by the UFPR ethics committee (protocol 23075.040498/2023-70).

Fecal samples were collected daily, with each animal placed individually in a 1 L beaker during collection. The feces were transferred into pre-weighed 1.5 mL tubes and weighed using an analytical balance. Each sample was then mixed with 1 mL of PBS buffer (137 mM NaCl, 2.7 mM KCl, 10 mM Na₂HPO₄, 1.8 mM KH₂PO₄) and briefly homogenized using a 1 mL pipette. The samples were then vortexed for 3 min and centrifuged at 500 x *g* for 2 min in a benchtop centrifuge. From the supernatant, 10 µL was used for the Nanoluc reaction, 100 µL was used for CFU counting and the remaining sample was stored at -80°C for later DNA extraction.

The Nanoluc reaction was performed using the NanoGlo Luciferase Assay System™ kit (Promega, USA), with the volume of the reaction mix matching the supernatant volume used. Luminescence was measured using a Synergy LX microplate reader (BioTek, USA) with a gain setting of 100. Raw luminescence values, expressed in relative luminescence units (RLU), were normalized using the following equation:

$$\text{Luminescence = }\frac{\text{RLU}}{{\text{(}\frac{\text{pDNA}}{\text{tDNA}}\text{)}}/\text{F}} \text{eq(3)}$$

where RLU represents relative luminescence units, pDNA is the mass of plasmid DNA in the sample, tDNA is the total mass of DNA extracted, and F represents the fecal mass in milligrams.

DNA extraction from the stored samples was performed using the Quick-DNA Fecal/Soil Microbe Microprep Kit (Zymo Research, USA), following the manufacturer's protocol. Before extraction, samples were thawed, centrifuged at 14,000 x *g* for 10 min at room temperature, and the supernatant was discarded. The pellet was used for DNA extraction.

For absolute plasmid quantification, 1 µL of extracted DNA was used for qPCR reactions, conducted with the Applied Biosystems™ PowerUp™ SYBR™ Green Master Mix kit (Thermo Scientific, USA) and the primers listed in Table S2. The reactions were run on an Applied Biosystems™ StepOnePlus thermocycler (Thermo Scientific, USA), with a standard curve generated for each reaction to allow for precise quantification (Figure S2). Cycling conditions were set according to the qPCR kit manufacturer instructions: 50°C for 2 minutes, 95°C for 2 minutes, followed by 40 cycles of 95°C for 15 seconds, 60°C for 15 seconds, and 72°C for one minute.

**Supplementary figures**


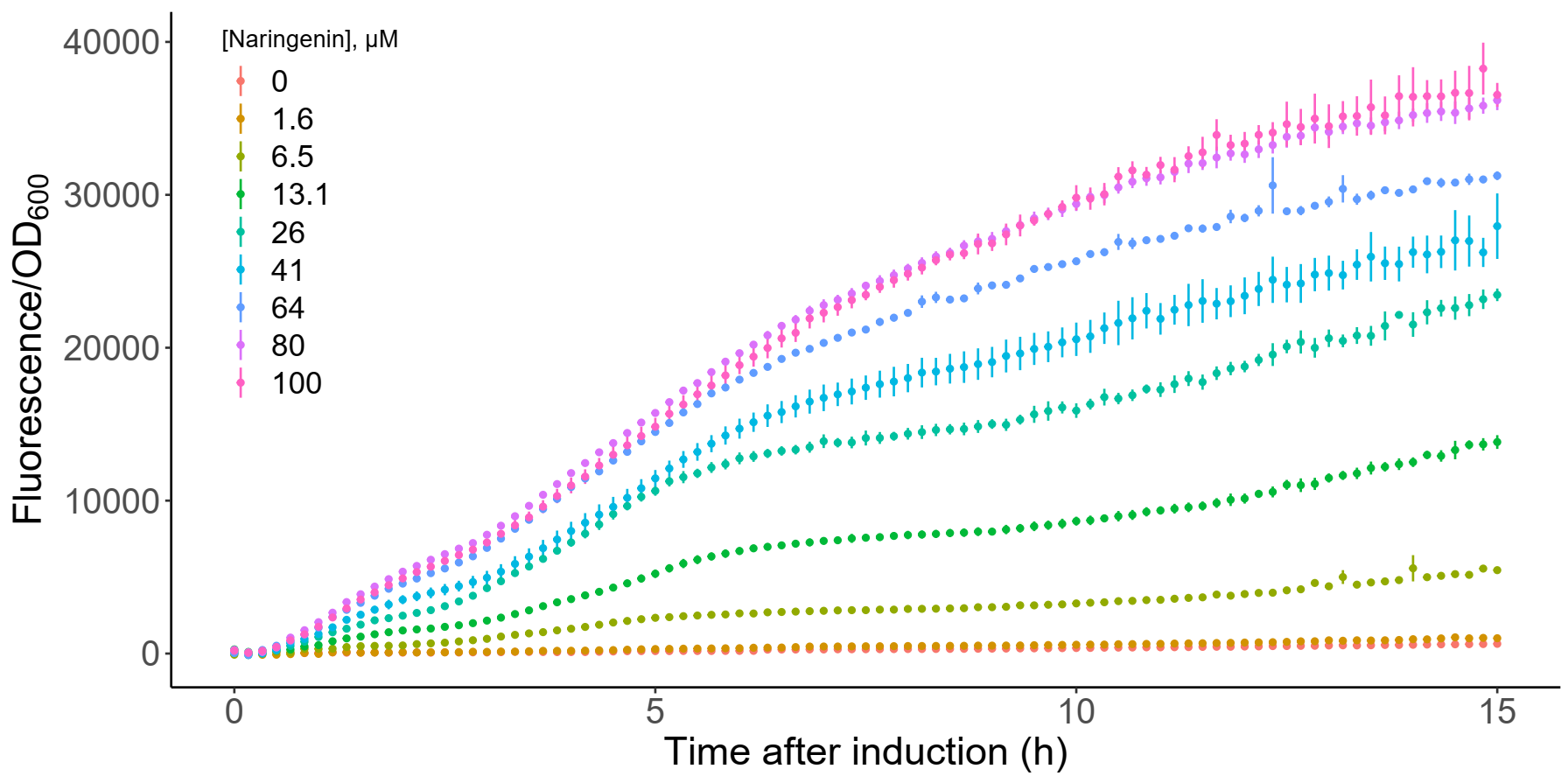


Figure S1. Time-course expression of sfGFP in response to different concentrations of naringenin. Points represent the average of three replicates, and error bars represent the standard error of the mean.

**
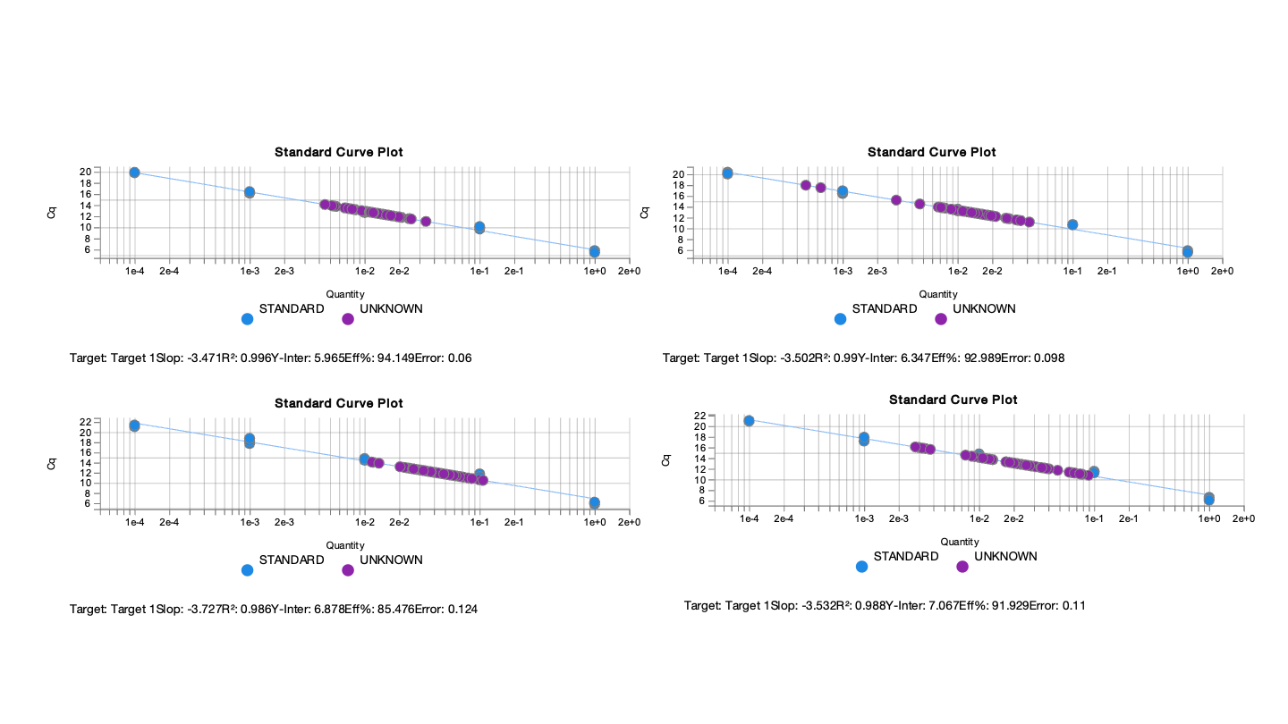
**

Figure S2. Plasmid quantification in the samples. Standard curves generated by qPCR from 10-fold serial dilutions of the 114-*fdeR*-*nanoluc* plasmid. The initial plasmid concentration was 1 ng/µL. The standard curve is shown in blue, and the fecal samples are shown in purple. Plots were generated using the Design and Analysis 2.7 software (Thermo Scientific).


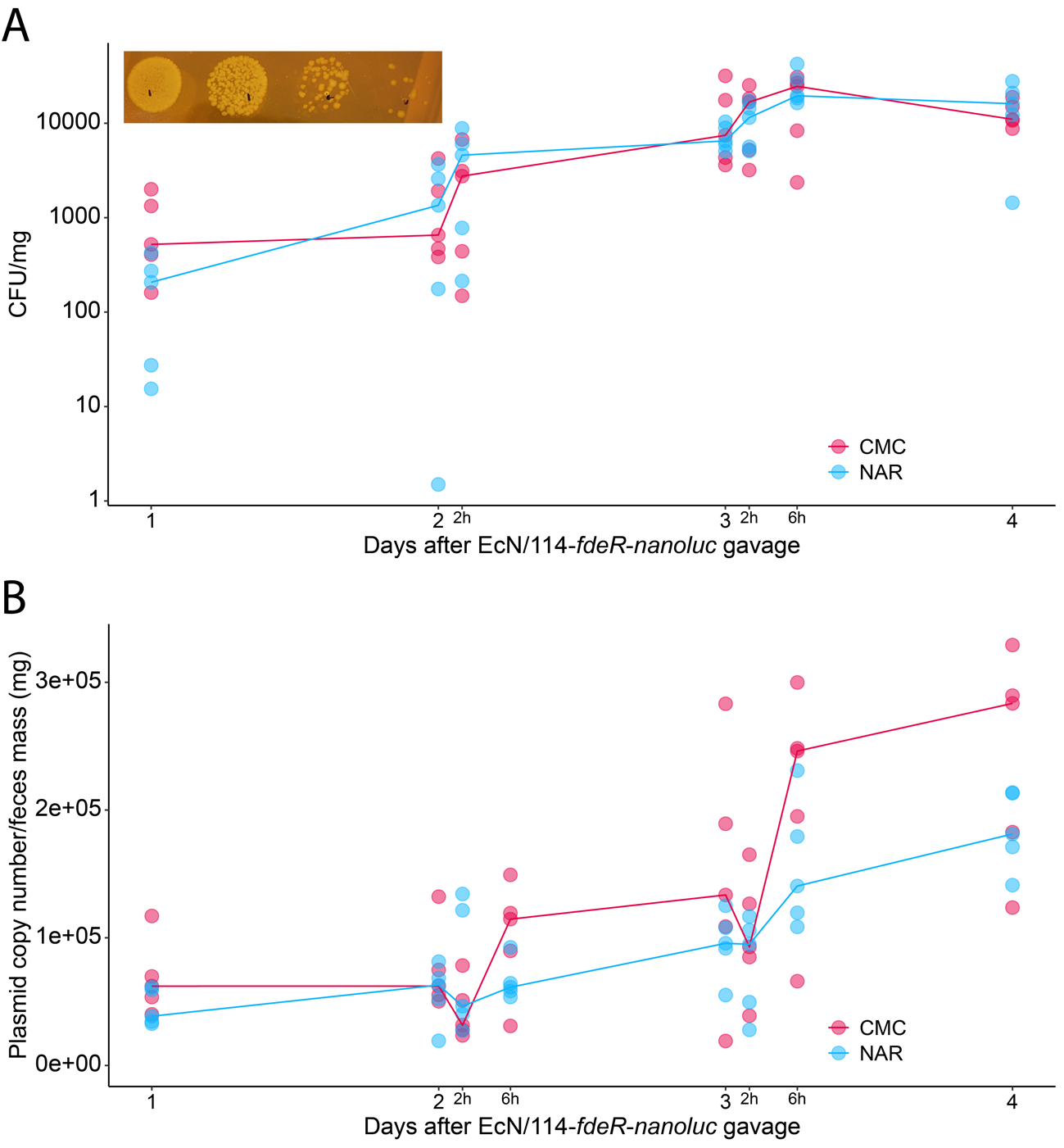


Figure S3. Colonization of mice gut by EcN/114-*fdeR-nanoluc*. A) Colony forming units per mg of feces. Colonies were obtained by serially diluting samples and plating 5 µL from each dilution into LB agar plates supplemented with 50 ug/mL of Kanamycin and 250 µg/mL of Ampicilin. Inset shows GFP expressing colonies from a representative replicate under blue light. B) Plasmid copy number in samples normalized by feces mass.

Table S1. Strains and plasmids used in this study.

| **Strain** | **Characteristics** | **Reference** |
| --- | --- | --- |
| *E. coli* Top10 | Cloning strain | Invitrogen (EUA) |
| *E. coli* Nissle 1917 | Wild-type strain |  |
| EcNλ::*sfgfp* | *E. coli* Nissle 1917 with chromosomal *sfgfp* insertion by CRIM (1, 3) | This study |
| EcN/114-*fdeR*-*nanoluc* | EcNλ::*sfgfp* containing the 114-FdeR-*nanoluc* plasmid | This study |
| **Plasmid** |  |  |
| 114-*fdeR*-*nanoluc* | Amp^R^, J23114-RBS-*fdeR*-T-P*_fdeA_*-RBS-*nanoluc*-T | This study |
| 114-*fdeR*-*sfgfp* | Amp^R^, J23114-RBS-*fdeR*-T-P*_fdeA_*-RBS-*sfgfp*-T | This study |

Table S2. qPCR primers used to quantify 114-*fdeR*-*nanoluc* in feces.

| ID | Sequence |
| --- | --- |
| fder-qpcr-fw | TCAAGGATGCGGTGCATGAT |
| fder-qpcr-rev | TCCGAAACCGAGATGCGAAA |

**Supplementary references**

1. Haldimann A, Wanner BL. 2001. Conditional-Replication, Integration, Excision, and Retrieval Plasmid-Host Systems for Gene Structure-Function Studies of Bacteria. Journal of Bacteriology 183:6384–6393.

2. Virtanen P, Gommers R, Oliphant TE, Haberland M, Reddy T, Cournapeau D, Burovski E, Peterson P, Weckesser W, Bright J, van der Walt SJ, Brett M, Wilson J, Millman KJ, Mayorov N, Nelson ARJ, Jones E, Kern R, Larson E, Carey CJ, Polat İ, Feng Y, Moore EW, VanderPlas J, Laxalde D, Perktold J, Cimrman R, Henriksen I, Quintero EA, Harris CR, Archibald AM, Ribeiro AH, Pedregosa F, van Mulbregt P. 2020. SciPy 1.0: fundamental algorithms for scientific computing in Python. 3. Nat Methods 17:261–272.

3. Ceroni F, Algar R, Stan G-B, Ellis T. 2015. Quantifying cellular capacity identifies gene expression designs with reduced burden. 5. Nat Methods 12:415–418.
